# Immune system activation and cognitive impairment in arterial hypertension

**DOI:** 10.1152/ajpcell.00219.2024

**Published:** 2024-11-04

**Authors:** Stefanie Schreiber, Philipp Arndt, Lorena Morton, Alejandra P. Garza, Patrick Müller, Katja Neumann, Hendrik Mattern, Marc Dörner, Jose Bernal, Stefan Vielhaber, Sven G. Meuth, Ildiko R. Dunay, Alexander Dityatev, Solveig Henneicke

**Affiliations:** ^1^Department of Neurology, Otto von Guericke University Magdeburg, Magdeburg, Germany; ^2^German Center for Neurodegenerative Diseases (DZNE), Helmholtz Association, Magdeburg, Germany; ^3^Center for Behavioral Brain Sciences (CBBS), Magdeburg, Germany; ^4^Department of Neurology, Heinrich Heine University Düsseldorf, Düsseldorf, Germany; ^5^Institute of Inflammation and Neurodegeneration, Otto von Guericke University Magdeburg, Magdeburg, Germany; ^6^Department of Cardiology, Otto von Guericke University Magdeburg, Magdeburg, Germany; ^7^Biomedical Magnetic Resonance, Faculty of Natural Sciences, Otto-von-Guericke University, Magdeburg, Germany; ^8^Department of Consultation-Liaison-Psychiatry and Psychosomatic Medicine, University Hospital Zurich, University of Zurich, Switzerland; ^9^Institute of Cognitive Neurology and Dementia Research (IKND), Otto-von-Guericke University, Magdeburg, Germany; ^10^Center for Clinical Brain Sciences, University of Edinburgh, Edinburgh, United Kingdom; ^11^Medical Faculty, Otto-von-Guericke University Magdeburg, Magdeburg, Germany

**Keywords:** arterial hypertension, cerebral small vessel disease, cognitive impairment, cytokines, immune system

## Abstract

Chronic arterial hypertension disrupts the integrity of the cerebral microvasculature, doubling the risk of age-related dementia. Despite sufficient antihypertensive therapy in still a significant proportion of individuals blood pressure lowering alone does not preserve cognitive health. Accumulating evidence highlights the role of inflammatory mechanisms in the pathogenesis of hypertension. In this review, we introduce a temporal framework to explore how early immune system activation and interactions at neurovascular-immune interfaces pave the way to cognitive impairment. The overall paradigm suggests that prohypertensive stimuli induce mechanical stress and systemic inflammatory responses that shift peripheral and meningeal immune effector mechanisms toward a proinflammatory state. Neurovascular-immune interfaces in the brain include a dysfunctional blood-brain barrier, crossed by peripheral immune cells; the perivascular space, in which macrophages respond to cerebrospinal fluid- and blood-derived immune regulators; and the meningeal immune reservoir, particularly T cells. Immune responses at these interfaces bridge peripheral and neurovascular unit inflammation, directly contributing to impaired brain perfusion, clearance of toxic metabolites, and synaptic function. We propose that deep immunophenotyping in biofluids together with advanced neuroimaging could aid in the translational determination of sequential immune and brain endotypes specific to arterial hypertension. This could close knowledge gaps on how and when immune system activation transits into neurovascular dysfunction and cognitive impairment. In the future, targeting specific immune mechanisms could prevent and halt hypertension disease progression before clinical symptoms arise, addressing the need for new interventions against one of the leading threats to cognitive health.

## Graphical abstract

**Figure:**
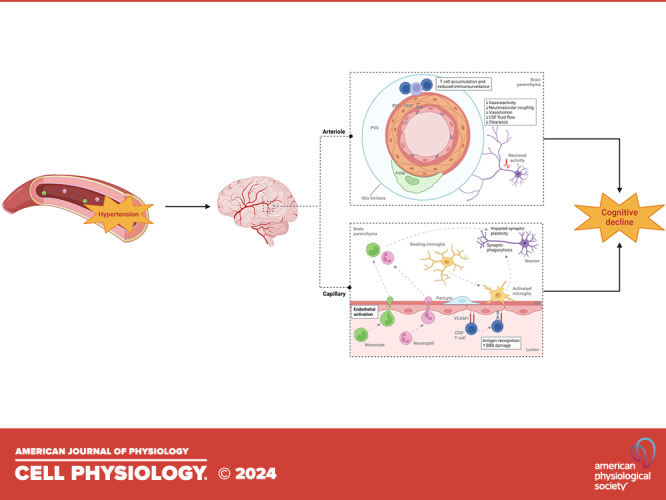

## INTRODUCTION

Arterial hypertension is a global health problem that affects more than 1.3 billion, that is, 30% of all adults worldwide (1). Arterial hypertension, particularly in midlife (here denoted as early disease stage), has been established as an independent modifiable risk factor for cognitive impairment and all-cause dementia, including vascular and Alzheimer-type dementia (AD) (2, 3). The Systolic Blood Pressure Intervention Trial (SPRINT) has shown, that an intense control of systolic blood pressure <120 mmHg—compared with standard systolic blood pressure lowering <140 mmHg—was associated with a 20% incidence reduction of mild cognitive impairment (MCI) (4).

In the brain, arterial hypertension is the main risk factor for sporadic cerebral small vessel disease (CSVD) and white matter hyperintensities of presumed vascular origin (WMH) (5). Dementia is directly related to both, WMH and arterial hypertension (6). Demyelination and axonal degeneration of white matter tracts are not only the substrate of WMH but also of cognitive impairment itself (7). The biological grounds directly linking arterial hypertension and dementia have indeed remained largely elusive.

Over the past decades, an activated immune system properly came into attention when deciphering hypertension pathophysiology (8, 9). Guzik and colleagues (9, 10) just very recently proposed a comprehensive paradigm of “meta-inflammatory mechanisms” at the multiorgan level, which are observed already very early in the course of disease and promote hypertension initiation and progression.

Importantly, despite sufficient blood pressure control or multidrug antihypertensive therapy, a significant proportion of individuals with arterial hypertension shows progression of CSVD (up to 50–60%), WMH, or cognitive impairment (11–13). Particularly, although the SPRINT has demonstrated that intense systolic blood pressure control may result in the maintenance of cognitive health in the short term, the results were inconclusive as to whether dementia itself will be delayed (4). This emphasizes the need for alternative targets for hypertension prevention and therapy, even beyond blood pressure reduction. These alternatives could be picked up through deciphering underlying inflammatory mechanisms of hypertension pathophysiology. Previous studies, however, have shown, that the use of nonspecific nonsteroidal anti-inflammatory drugs, for example, offered only modest protection from AD and did not prevent AD-related or vascular cognitive decline (14, 15). Hence, the modification of immune mechanisms in arterial hypertension will probably need more selective and targeted approaches.

In this review, we not only focus on the activated immune system in arterial hypertension and its impact on the brain and cognition. We further develop a temporal concept that presents brain inflammatory endotypes and their significance along the course of hypertension. We lay a conceptual ground for future targeted strategies that could intervene at various and individualized preclinical timepoints during a long window of opportunities for the prevention of cognitive impairment and dementia in arterial hypertension.

## SYSTEMIC IMMUNE SYSTEM ACTIVATION IN ARTERIAL HYPERTENSION

Guzik et al. (9) summarized the evidence that early immune system activation contributes to the manifestation and chronic exacerbation of arterial hyplertension. The overall concept suggests the existence of a “neuroimmune axis,” where chronic prohypertensive stimuli systemically activate the sympathetic nervous system (SNS) and the renin-angiotensin-aldosterone system (RAAS). Mechanical stress together with the SNS activates immune cells (e.g., splenic T cells, B cells, macrophages, circulating monocytes) and the release of upstream inflammatory regulators [e.g., cytokines, chemokines, damage-associated molecular patterns (DAMPs), complement system activation products], overall promoting a systemic, that is, downstream, immunological response (16–18). This concept mirrors the recently discovered neuroimmune (cardio)vascular interfaces, where the nervous, immune, and vascular systems interact to promote vascular wall remodeling in large artery disease, that is, atherosclerosis, and, in turn, to accelerate vascular aging (19).

Innate and adaptive immune cells are involved in the complex interplay that modulates immune mechanisms in hypertension. Monocytes, natural killer (NK) cells, and T cells traffic from immune organs and infiltrate the (peri)vascular walls, and—together with mechanical stress—they locally trigger the release of proinflammatory cytokines and chemokines. Key immune regulator proteins include interleukin-6 (IL-6), IL-1β, tumor necrosis factor α (TNFα), and interferon γ (IFN-γ) or C-X-C motif ligand 5 (CXCL5) and CXCL2 (20), which further recruit and activate neutrophils or adaptive immune cells, such as T and B cells. Activated CD4^+^ T cells can differentiate into effector T helper cells (e.g., T_H_1, T_H_17 subtypes), which—again—secrete cytokines, for example, proinflammatory IL-17, IL-6, TNFα, or IFN-γ, that foster perivascular inflammation (21). Inflammation together with mechanical stress, that is, shear stress, impacts the plasticity of vascular smooth muscle cells (VSMC) and modulates the VSMC phenotype. The macrophage-like VSMC phenotype, for example, produces different cytokines (e.g., IL-1β), chemokines (e.g., CC-chemokin-ligand-2, CCL2), and adhesion molecules itself, further promoting chronic vascular inflammation and facilitating ongoing immune cell recruitment (22).

CD4^+^ T cells can also convert into regulatory T cells (T_reg_ cells) as well, which, conversely, counteract immune cell activation (e.g., effector T cells), inflammation (e.g., IL-17 increase), and vascular dysfunction. T_reg_ cells have a critical role in lowering the proinflammatory response and are known as key players in the resolution of inflammation, needed for promoting tissue repair and restoring immune microenvironment balance (23, 24). T_reg_ cell deficiency is related to hypertension development and severity and microvascular injury in hypertensive mice (25). Conversely, in hypertensive rodents transfer of adoptive T_reg_ cells lowers blood pressure and attenuates vascular stiffness in hypertensive mice (26), pointing toward resolution of inflammation as a possible target for treating hypertension. In arterial hypertension, a suspected disbalance of proinflammatory and protective inflammatory pathways will need further elaboration.

These lines of evidence that relate systemic to vascular inflammation have predominantly derived from studies in hypertensive rodents with a focus on large vessels, particularly aortic atherosclerosis (9).

**Figure 1.**
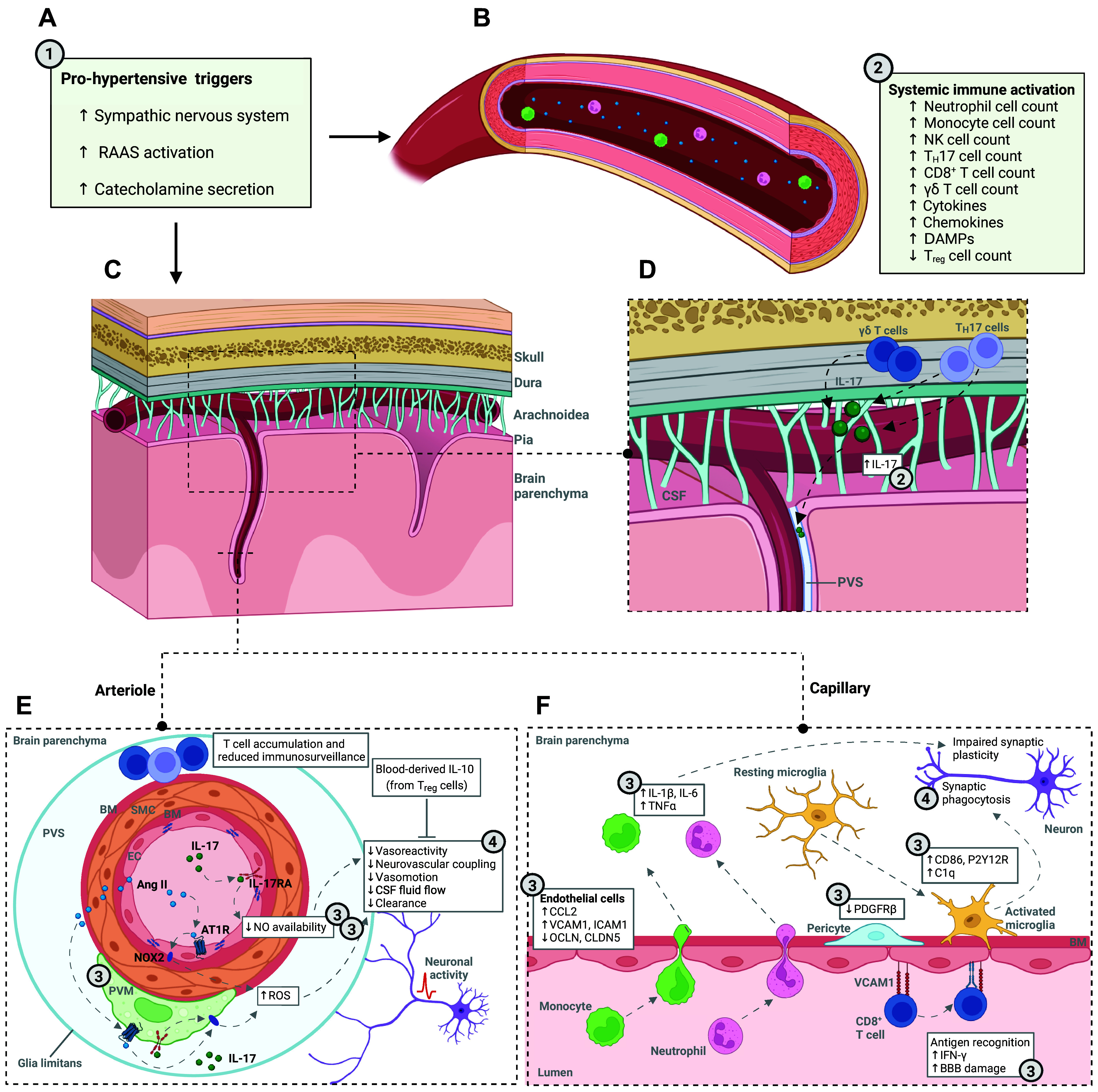
Temporal framework and interactions at neurovascular-immune interfaces in the pathogenesis of arterial hypertension. The figure illustrates interactions of systemic inflammatory responses in arterial hypertension at neurovascular-immune interfaces with the following temporal framework: ① peripheral and meningeal immune system activation; ② induction of peripheral and meningeal immune system effector mechanisms; ③ which bridge the interaction between immune system, neurovascular unit, and glial inflammation, to ④ act on brain perfusion, (perivascular) clearance of toxic metabolites and synaptic and neuronal dysfunction, which underlie cognitive impairment. *A*: prohypertensive triggers comprise chronic activation of the sympathetic nervous system and the renin-angiotensin-aldosterone system (RAAS), and increased secretion of catecholamines. *B*: these stimuli promote local inflammation and mechanical stress and lead to an activation of the immune system with recruitment of immune cells into the systemic circulation and a shift toward proinflammatory immunological regulators [cytokines, damage-associated molecular patterns (DAMPs)]. *C*–*F*: cellular and molecular changes of cerebral (micro)vessels in arterial hypertension, their interaction with immune cells and immunological regulators, and consequences for microglia and synapses/neurons. *C*: overview of the cranial/meningeal layers and brain vasculature. *D*: resident meningeal T helper cells (T_H_17 cells) and γδ T cells secrete their main effector cytokine interleukin 17 (IL-17) into the cerebrospinal fluid (CSF) circulation, from where it reaches the perivascular space (PVS) of pial and penetrating arterioles. *E*: penetrating arteriole within the PVS: blood-derived immune regulators induce *1*) endothelial IL-17/IL-17RA signaling, which inhibits endothelial nitric oxide (NO) synthase and *2*) Ang-II/AT1R/NOX2 signaling in perivascular macrophages (PVM) producing reactive oxygen species (ROS). Both NO synthase inhibition and ROS impair endothelial vasodilation during neuronal activity, that is, neurovascular coupling. Neurovascular dysfunction can be rescued by IL-10 derived from regulatory T cells (T_reg_ cells). *F*: the blood-brain barrier (BBB): in response to arterial hypertension, BBB integrity is impaired due to endothelial downregulation of tight junction gene expression (occludin, *OCLN*; claudin-5, *CLDN5*) and pericyte injury allowing peripheral preactivated immune cells to immigrate and secrete proinflammatory cytokines. Gene expression of adhesion molecules [e.g., vascular cell adhesion molecule 1 (*VCAM1*), intercellular adhesion molecule 1 (*ICAM1*)], chemokine secretion [e.g., CC-chemokin-ligand-2 (*CCL2*)], and presentation of brain-derived antigens by vascular cells activate CD8^+^ T cells, which can disturb BBB integrity. Microglia are activated and recruited to the vasculature and are able to phagocytose adjacent synapses. Created with BioRender.com. Ang-II, angiotensin II; AT1R, angiotensin II receptor type 1; BM, basement membrane; C1q, complement component 1q; EC, endothelial cell; IFN-γ, interferon γ; NOX2, NADPH oxidase 2; NK cells, natural killer cells; PDGFRβ, platelet-derived growth factor receptor β; SMC, smooth muscle cell; TNF, tumor necrosis factor.

Human biofluid studies of blood from patients with arterial hypertension in mid- or at most early later life, which commonly did not reveal hypertensive end-organ damage, support the framework of an early systemically activated immune system. Circulating cell frequencies of, for example, neutrophils, proinflammatory intermediate and nonclassical monocytes, NK cells or T_H_17 cells are increased, cytokine levels of IL-17, IL-6, and TNFα are elevated, and anti-inflammatory T_reg_ cell frequencies, producing IL-10, are decreased (9, 18, 27–30) (Fig. 1, *A* and *B*).

## MENINGEAL IMMUNITY IN ARTERIAL HYPERTENSION

Recent findings also highlight meningeal immunity as a critical site of immune responses in experimental hypertension with special emphasis on meningeal T cells. In deoxycorticosterone (DOCA)-salt-sensitive hypertension, at initial disease stages, circulating T_H_17 and γδ T cells are increased and migrate from the periphery into the dura. Although the total number of meningeal T cell subpopulations remains unchanged, their expression and secretion of proinflammatory IL-17 into the cerebrospinal fluid (CSF) is elevated (31). These data suggest that in hypertension, peripherally preactivated T cell subpopulations are able to migrate into the dura and secrete immune regulators into the CSF circulation. This adds another layer of complexity to the origin of immune regulators in the brain.

## NEUROVASCULAR UNIT IN ARTERIAL HYPERTENSION

There is scarce knowledge about the cross talk between peripheral immune system activation and the brain in arterial hypertension, and several considerations presented here rely on results mainly derived either from in vitro studies, experimental models focusing on the extracerebral larger arteries, particularly aorta, or from nonhypertensive disease, for example from experimental models of multiple sclerosis.

The neurovascular unit (NVU) is composed of neurons, perivascular astrocytes, microglia, perivascular macrophages (PVM), pericytes, and endothelial cells, which share complex signaling networks, and are together responsible for the maintenance of the blood-brain barrier (BBB) and cerebral homeostasis, as well as cerebral blood flow (CBF) control (32).

In arterial hypertension, several processes might interact at the NVU. Overall, mechanical stress affects endothelial signaling pathways involved in myoendothelial coupling, that is, hemodynamic response, for example, through altered nitric oxide (NO) production and bioavailability in meningeal or larger cerebral arteries and altered K+ conductivity in parenchymal arterioles and capillaries (33, 34). Mechanical stress further acts on the endothelial barrier function and on the endothelial secretome, which comprises the endothelial expression of adhesion molecules, inflammatory mediators, and the extracellular matrix (ECM) (35). Furthermore, inflammatory mediators, such as different cytokines, promote the NVU’s upregulation of the mechanosensitive channel Piezo 1, sensing the unit’s structures against mechanical stress (36). Peripheral leukocytes become attracted, adhere to the endothelium and to pericytes, and traffic into the brain. In parallel, peripheral immune system activation itself, as outlined above, promotes changes at the stressed NVU and facilitates NVU—immune cell—interactions. At early hypertension, NVU elements act dynamically and seem to balance pro- and anti-inflammatory properties. These early alterations might be compensatory and could be reversed upon prevention and targeted therapies. Over time, peripheral immune cells that reach the brain parenchyma, together with microglia and astrocytes, might maintain reciprocal activation through the secretion of cytokines or generation of proteolytic fragments, promoting increased leukocyte infiltration and resulting in chronic inflammation.

The following sections will outline the processes in more detail and will demonstrate, how they foster—presumably irreversible—structural alterations during the longer course of arterial hypertension. Figure 1, *C*–*F*, visualizes these mechanisms.

## BRAIN ENDOTHELIAL CELLS IN ARTERIAL HYPERTENSION

In contrast to the peripheral vasculature, brain endothelial cells form tight junctions to prevent the paracellular passage of leukocytes. During homeostasis, brain endothelial cells express low levels of adhesion molecules and inflammatory mediators, such as chemokines or cytokines, contributing to the brain’s specialized immune regulatory environment (37).

In spontaneously hypertensive stroke-prone rats (SHRSP), expression of tight junction proteins, such as occludin or claudin 5, is reduced (38). Furthermore, in hypertension peripheral inflammatory mediator proteins, such as TNFα or INF-γ, local mechanical stress and glial activation (see glial inflammation in arterial hypertension) together *1*) promote the expression of endothelial adhesion molecules, for example, vascular cell adhesion molecule 1 (VCAM1) or *2*) promote endothelial cytokine secretion (e.g., IL-6), and *3*) alter capillary endothelial K+ conductivity (39, 40). Inflammatory cytokines released by activated endothelial cells additionally induce the macrophage-like VSMC phenotype and VSCM proliferation. These processes in sum reduce autoregulatory properties, which in turn results in increased mechanical/shear stress and ongoing endothelial dysfunction/activation (22). Systemically circulating IL-17 acts on its activated endothelial receptor (IL-17RA), which further impairs endothelial vasodilation/autoregulation, for example, through reduced NO bioavailability of cerebral endothelial cells (8, 31, 41, 42) (Fig. 1*E*). Although T_H_17 cells are major producers of IL-17, additional immune cell sources include γδ T cells and NK cells.

Activated endothelial cells have the potential to present antigens to naive CD8^+^ or CD4^+^ T cells, promoting the T cells’ activation, proliferation, and differentiation, for example, into T_H_17 or Treg cells. Strikingly, upon activation, brain endothelial cells could also present abluminal, that is, brain-derived, antigens to blood CD8^+^ T cells. Once activated, these CD8^+^ effector T cells disturb endothelial layer integrity, for example, through the disruption of cadherin junctions (43), promoting leucocyte infiltration into the brain (38). Subsequent BBB leaks with perivascular fibrinogen clustering further foster antigen-presentation-mediated luminal and abluminal T cell activation and recruitment (44).

Correspondingly, in spontaneously hypertensive rats (SHR), T and NK cells adhere to brain microvascular endothelial cells with upregulated *VCAM1* (33). In contrast, in the normotensive rat brain T cells mainly distribute in the meninges and choroid plexus. Notably, intravital microscopy experiments support that neither homeostatic nor activated lymphocytes undergo interactions with brain microvessels in normotensive rodents unless endothelial cells are preactivated through, for example, systemically administered TNFα (45) (Fig. 1*F*).

Experimental data are further supported by a human study that administered plasma from asymptomatic CSVD with WMH to human brain endothelial cultures. Upon exposition, endothelial signaling pathways, including *CXCL8* and *IL-12A* gene expression (both of them are chemoattractants for neutrophils), became activated (46).

## BRAIN PERICYTES IN ARTERIAL HYPERTENSION

Brain pericytes have a pivotal role at the interface between the peripheral immune system and the brain, influencing leucocyte recruitment and microglia polarization. Through their perivascular localization, they work closely with endothelial cells to maintain BBB integrity and regulate local CBF (47).

Similar to endothelial cells, pericytes respond to (peripheral) cytokines, such as IL-6 or TNFα, by modifying their secretome, which includes the secretion of chemokines, for example, CC, CX3, CXC3 chemokine subfamilies, and the expression of adhesion molecules. Chemokines and adhesion molecules attract and guide leucocytes into the brain, that is, monocytes, neutrophils, or T cells, and promote their binding to pericytes and ultimately their transmigration into the brain [extensively reviewed in Ref. (47)]. Transcriptomic research in early-stage hypertensive rodents has shown that pericytes upregulate integrins and VCAM1, that are key for the interaction with immune cells (48). These interactions between pericyte/endothelial adhesion molecules and leucocytes (mainly neutrophils) contribute to capillary stalling, which is a common finding in the brain of hypertensive rodents, and further impairs autoregulation and cerebral blood flow (49–51).

Furthermore, once activated, pericytes can promote T_reg_ formation and secrete several pro- or anti-inflammatory factors themselves. For example, IL-6, TNFα, IFN-γ, and IL-1β, or CX3CL1 and IL-33, which in turn can induce or prevent proinflammatory endothelial or glial activation.

Pericyte activation by TNFα and IFN-γ, for example, can conversely also enhance compensatory phagocytotic capacities and internalization of neurotoxic blood-derived products, whereas transforming growth factor 1β (TGF-1β), on the contrary, attenuates the phagocytotic uptake by pericytes.

Hence, pericytes have the potential to sense and respond to peripheral inflammation and to propagate it through the NVU into the perivascular and brain parenchyma (for review, see Ref. 47). As stated, pericytes can thus be considered key elements for the cross talk between the peripheral immune system and neuroinflammation in arterial hypertension, and this cross talk most likely takes place already at early disease stages (52). Indeed, similar to circulating immune system activation, in arterial hypertension, there is a lack of understanding of a suspected disbalance of proinflammatory and protective inflammatory pathways for NVU structure and function as well.

Chronic pericyte exposure to TNFα, IFN-γ, or IL-1β (e.g., during the longer course of hypertension) compromises pericyte function, leading to BBB disruption, which is mediated through the decrease of angiopoietin 1 or platelet-derived growth factor receptor β (PDGFRβ) and its signaling (53, 54).

Human autopsy studies in patients with longer-lasting hypertension likewise reported pericyte injury, that is, lower parenchymal levels of PDGFRβ, together with BBB leaks, that is, parenchymal fibrinogen deposition (55). In one human postmortem study in patients with WMH and vascular dementia or AD, even the density of pericytes was reduced (56). Unpublished data additionally decidedly point toward diminished pericyte density in human CSVD brains (see “Blood-Brain Barrier Dynamics in Vascular Dementia: Unravelling the Tripartite Crosstalk between the Endothelium, Pericytes, and Microglia - Oxford Talks”) (Fig. 1*F*).

As a promising biomarker, soluble PDGFRβ (sPDGFRβ) accumulates in the human CSF upon pericyte injury and is associated with hippocampal BBB breakdown and early cognitive impairment, independent of AD pathology (57) (Fig. 1*F*). However, there are no studies so far that evaluated sPDGFRβ in patients with early-stage hypertension or early-stage hypertensive CSVD/WMH.

## GLIAL INFLAMMATION IN ARTERIAL HYPERTENSION

Microglia, PVM, and astrocytes are found closely opposed to the outer vascular wall of parenchymal vessels. PVM are particularly located within the perivascular space (PVS) of pial and parenchymal arterioles/venules, sitting between the basal membrane of VSMC and the glia limitans formed by astrocyte endfeet (Fig. 1*E*).

In arterial hypertension, several processes promote glial and PVM activation. Peripheral and local factors, such as IFN-γ, IL-10, or TGF-β (e.g., from activated endothelium or glia), interact with microglia and can foster glial activation (58).

Furthermore, reduced pericyte coverage and larger BBB leaks allow the extravasation of toxic blood-derived products, such as fibrinogen. Subsequently, perivascular fibrinogen/fibrin accumulation and consecutive CCL2 secretion from, for example, activated endothelial cells, pericytes, or fibroblasts as well as leucocyte infiltration promote microglia activation, migration toward the brain microvasculature, and perivascular clustering (59–61).

In detail, in a rodent model at stages comparable to midlife hypertension, that is, early disease, microglia were activated and—compared with age-matched normotensive controls—the expression of different surface proteins (e.g., CD11b/c, CD86, P2Y12R, CD200R, major histocompatibility complex II) were increased. Ang II further promotes microglial activation through stimulation of Toll-like receptor (TLR) 4 on the microglia surface (62). Results indicate a dynamic microglial phenotype, with proinflammatory properties (CD11b/c, CD86), as well as chemotactic and anti-inflammatory responsivity as reflected by P2Y12R and CD200R upregulation (33, 38). P2Y12R is essential for contacts between microglia and endothelial cells, local vasoreactivity, and BBB repair. Its upregulation in hypertension might be a protective mechanism of vessel-associated microglia subpopulations (63). Microglial CD86 and major histocompatibility complex II expression may promote further (luminal and abluminal) T cell activation (38).

Dynamic microglial phenotype persisted at later stages of hypertension, that is, longer lasting disease, but expression pattern shifted toward more proinflammatory properties (for example, elevation of CD11b/c and decrease of CD200R, CX3CR1; each compared with age-matched normotensive controls) (38).

Once activated, microglia could secrete cytokines, such as IL-1α, TNFα, and complement component 1q (C1q), that—in turn—induce astrocyte activation (64), as it has been shown in experimental longer-lasting arterial hypertension (33).

PVM contribute to the manifestation of experimental hypertension. Elevated levels of circulating IL-1β in hypertension lead to prostaglandin E2 overexpression in PVM, which excites neurons in hypothalamic paraventricular nuclei (via EP3 receptor signaling) and increases SNS activity (65). Furthermore, located in the PVS, macrophages respond to blood- or CSF-derived immune regulators with cellular activation and production of reactive oxygen species (ROS), promoting microvascular oxidative stress (31, 66) (Fig. 1, *D* and *E*).

Human autopsy data from patients with chronic arterial hypertension and WMH were in line with the experimental framework and showed activated glial cells, clustering around the brain microvasculature. For example, microglia displayed increased proinflammatory CD68 expression, whereas astrocytes showed glial fibrillary acidic protein upregulation (33, 38, 67–69).

## TEMPORAL FRAMEWORK: FROM IMMUNE SYSTEM ACTIVATION TO COGNITIVE IMPAIRMENT IN ARTERIAL HYPERTENSION

Based on the presented paragraphs we propose that in arterial hypertension, the complex development from immune system activation to brain pathology and subsequent cognitive impairment relies on different, presumably, overlapping temporal stages: *1*) peripheral and meningeal immune system activation, *2*) induction of peripheral and meningeal immune system effector mechanisms, which *3*) bridge the interaction between immune system, NVU, and glial inflammation, to *4*) for example, act on brain perfusion, (perivascular) clearance of toxic metabolites and synaptic and neuronal dysfunction, which together are substrates that underlie cognitive impairment [modified from (9)].

Figure 1 summarizes the presumed temporal framework, in which prohypertensive stimuli induce mechanical stress and systemic and meningeal inflammatory responses with immune cell activation ① and a shift toward proinflammatory immune regulators ②. Neurovascular-immune interfaces, where the brain and the immune system interact, include *1*) a dysfunctional BBB, crossed by peripheral immune cells, *2*) the PVS, in which PVM respond to CSF- and blood-derived immune regulators, and *3*) the meningeal immune reservoir, particularly T cells ③. Interactions at those interfaces disturb the immune-privileged state of the brain and contribute to impaired perfusion, clearance, and synaptic plasticity, which—together—mediate the effect of immune system activation on cognitive impairment ④.

## BRAIN PERFUSION, CLEARANCE OF TOXIC METABOLITES, AND SYNAPTIC PLASTICITY IN ARTERIAL HYPERTENSION

The mechanisms that could link an activated immune system to cognitive impairment in arterial hypertension—presumably even in the still absence of downstream pathologies such as (pronounced) WMH—are manyfold. Here, we consider alterations of brain perfusion, dysfunction of the waste clearance system, and direct interactions between the immune system and synapses or neurons as central mechanisms in hypertension.

Experimental and human studies in this context have predominantly been focusing on the hippocampus and fronto-parietal cortex, mainly, because these regions might be particularly susceptible to hypertension-related cognitive impairment. Hypertensive rodents, for example, commonly show learning and memory deficits, regardless of the form of arterial hypertension (70–72). To this effect, human studies have related hypertension to alterations in memory, executive function, and attention (73–75). For the hippocampus, its pivotal involvement in hypertension-related cognitive impairment is particularly supported by the commonly observed relationship between brain arteriolosclerosis (as end-stage pathology in hypertension) and hippocampal-specific late-life (neuro)degenerative pathologies. For example, limbic-predominant age-related TDP-43 encephalopathy, primary age-related tauopathy, or hippocampal sclerosis (76–78).

With regard to brain perfusion, several studies report an association between immune system activation in experimental arterial hypertension and CBF reduction, mostly mediated through endothelial/NVU function loss, which in turn promotes cognitive impairment. For example, brain infiltration of CD8^+^IFN-γ^+^ T cells and reduced microvascular pericyte density are related to global grey matter CBF decline in experimental aortic constriction, which induces chronic pressure overload (79). On the cellular level, angiotensin II (Ang-II)/Ang-II receptor type 1 (AT1R) or IL-17/IL-17RA signaling reduces endothelial NO bioavailability, impairing endothelial-mediated vasodilation in salt-sensitive and Ang-II-induced hypertension (31, 42, 80). Conversely, neutralization of IL-17, inhibition of its receptor, or elimination of its cellular sources improves neurovascular coupling and endothelial vasodilation, despite elevated blood pressure (31, 41). Furthermore, infusion of T_reg_ cells and their corresponding secretion of anti-inflammatory IL-10 is able to rescue CBF reduction, most likely through lowering systemic inflammation (80) (Fig. 1*E*).

(Perivascular) clearance routes emerged as essential mechanisms of brain homeostasis through the removal of metabolites along meningeal lymphatic vessels into the deep cervical lymph nodes. Perivascular clearance is driven by small vessel wall constrictions (e.g., arterial pulsatility or vasomotion) (81, 82). In Ang-II-induced hypertension, SHR, and SHRSP, perivascular flow speed is reduced by ∼40%, which has been mainly attributed to a blood pressure-related loss of flexibility and wall motility—and thus—remodeling of the cerebral microvasculature (during ongoing disease course) (81, 83, 84). Indeed, it is conceivable that immune system alterations at early disease stages might contribute to the dysfunction of brain clearance as well.

During homeostasis, that is, normotension, PVM in the PVS, together with mural cells (pericytes, VSMC), regulate perivascular fluid flow (85). In Ang-II-induced and DOCA salt-sensitive hypertension, PVM become dysfunctional, which could affect perivascular fluid dynamics. PVM are activated through circulating Ang-II that enters the PVS when BBB integrity decreases and binds to the macrophages’ AT1R. PVM-activation is further fostered through CSF-derived IL-17, which is produced by resident meningeal γδ T cells and TH17 cells and binds on the PVM's IL-17RA. Both signaling pathways lead to NADPH oxidase 2 (NOX2)-derived production of large amounts of ROS, which in turn impair vasodilation and neurovascular coupling. Blockage of AT1R or IL-17RA signaling, on the contrary, improves vasodilation and neurovascular coupling in long-term hypertension (31, 66, 86). Overall, neurovascular coupling is tightly connected to perivascular CSF flow and, thus, crucial for the clearance of metabolic waste (87, 88). PVM (function) loss has further been related to decreased ECM degradation (through reduced production of matrix metalloproteinases), accumulation of neurotoxic waste products, and synaptic dysfunction, underlining the macrophages’ role in sustaining brain homeostasis (85). Importantly, ECM could accumulate in the PVS, potentially hindering perivascular clearance (89). A weakened driving force of perivascular fluid flow does not only influence waste clearance but also immune cell trafficking within perivascular conduits. During physiological conditions, lymphocytes are recruited from the CSF or the bloodstream, actively surveil PVS, and interact with PVM. If immune cell trafficking dynamics are disturbed, cells accumulate and prolonged presentation of brain-derived antigens by PVM might induce T cell activation and autoimmunization (reviewed in Ref. 90).

In addition, impaired synaptic plasticity and function, presumably predominantly caused by inflammatory regulators, for example, cytokines, or resident immune cell activation, for example, microglia, can be considered an additional route to cognitive impairment in arterial hypertension.

Although low physiological levels of proinflammatory cytokines, such as IL-1β, TNF-α, IL-17, and IFN-γ, support physiological plasticity and homeostatic regulations in neural networks, their elevation is detrimental during uncontrolled inflammatory processes (91). Acting through cognate neuronal receptors, they may activate p38 MAPK and impair synaptic plasticity and different forms of memory, and destabilize actin cytoskeleton in dendritic spines. In contrast, anti-inflammatory cytokines IL-4 and IL-10 promote synaptic plasticity and brain network dynamics through a dual mechanism: by dampening the overactivation of the immune system and by direct modulation of synaptic function in neurons.

For example, downregulated synaptic markers, reduced synapse density, and impaired synaptic plasticity are detected in cortical regions and the hippocampus of SHRSP as well as in Ang-II-induced hypertensive mice (92, 93). In parallel, leukocytes migrate into the brain and complement products (e.g., *C1q*) are upregulated (93). In this context, microglial phagocytosis in a C1q-dependent manner and subsequent lysosomal digestion is increasingly recognized as a mechanism underlying the synapse loss, as it has—so far—mainly been shown in neurodegenerative diseases (8, 94, 95).

In addition, activation of microglia and astrocytes following BBB leakage may lead to degradation of ECM of perineuronal nets (PNN) associated with fast-spiking parvalbumin-expressing interneurons and accumulations of perisynaptic ECM around excitatory synapses on excitatory neurons (96). Loss of PNN in turn may result in impaired precision of hippocampal memories (97) and open a window for neural network remodeling, whereas accumulation of perisynaptic ECM may amplify neuroinflammatory effects through local accumulation of inflammatory messengers, further constraining synaptic plasticity and homeostatic mechanisms and contributing to stabilization of pathological network configurations.

Considering human data, arterial hypertension has likewise been related to reduced brain perfusion (including decreased vascular reactivity) and synaptic/neuronal dysfunction, which seem to mediate the relationship between hypertension and cognitive function [for example, Refs. (12, 98–100)].

There is further emerging evidence for an association between hypertension and the integrity of the brain clearance system, while it remains under debate whether this relationship impacts cognition (101–103). For example, human studies associated hypertension consistently with PVS severity, and genome-wide association studies (GWAS) proposed a causal relationship (104, 105). From a functional point of view, another study found that the removal of an intravenously administered tracer was reduced in participants with CSVD, most of whom were hypertensive (106). Furthermore, GWAS related PVS severity to several (immune) processes that are affected in arterial hypertension as well, for example, brain endothelial dysfunction with reduction of tight junctions and impaired BBB integrity (105).

However, to the best of our knowledge, no human study so far has focused on hypertensive participants to shed light on the role of the activated immune system on cognition. Particularly, there is no data, on whether and to what extent this relationship could be mediated by nondownstream (i.e., presumably early-disease) hypertensive brain pathologies, such as alterations of CBF, clearance, or synaptic plasticity.

## CONCLUSION AND FUTURE PROSPECTIVE

We provide a comprehensive overview that demonstrates how an activated immune system interacts with disease initiation and progression in arterial hypertension. We present, how hypertension could lead to cognitive impairment, particularly taking into account the interplay between peripheral and brain immune systems at different neurovascular-immune interfaces. Our framework, which considers a temporal perspective on disease progression, assumes an early activation of peripheral and meningeal immune cells, that provoke—potentially—reversible and dynamic endothelial, pericyte, and glial alterations. Longer-lasting arterial hypertension disturbs brain homeostasis and promotes different mechanisms, such as impaired perfusion, clearance, and synaptic plasticity, that—together—pave the way to cognitive impairment.

One has to consider this framework as a hypothetical construct that has derived from the in-depth review of several independent, mainly experimental, studies. Indeed, the proof of the sequential development of the different stages is still missing and demands longitudinal studies that cover the course of disease within the same animal.

Naturally, as said, most of the available evidence for this framework results from experimental models of hypertension. Initiating or early (immunological) disease stages in humans have, on the contrary, nearly not been elaborated, particularly not with respect to the brain. Human data instead mainly stem either from few autopsy studies or from patients with longer-lasting arterial hypertension and—correspondingly—already existent or even extended downstream brain pathologies, such as WMH.

In Tables 1 and 2, we hence provide a summary of existing human studies that have investigated systemic immune markers in the blood in different cohorts suffering from arterial hypertension and/or CSVD diagnosed through magnetic resonance imaging (MRI). Cross-sectionally, immune cells, particularly circulating neutrophils and monocytes, were in several of the studies related to larger WMH volume. Longitudinally, baseline neutrophil-to-lymphocyte ratio and frequencies of neutrophils and monocytes were likewise associated with the progression of WMH. One study reported an association between levels of the microglial activation marker soluble triggering receptor expressed on myeloid cells 2 (sTREM2) in blood plasma and greater WMH (107). In addition, baseline sTREM2 in CSF was associated with WMH and overall CSVD progression (108). Conversely, circulating blood cytokines did not show consistent cross-sectional associations with WMH and/or CSVD severity. However, one longitudinal immunophenotyping study revealed IL-6 and IL-17 at baseline as predictors of WMH progression after nine years (109).

**Table 1.** Cross-sectional relationship between systemic immune markers and neuroimaging markers of cerebral small vessel disease

Study	Mean Age	% HTN	% Stroke	% LI	% CMB	MRI	∼CSVD	∼WMH	∼LI	∼CMB	Reference
*Neutrophil-to-lymphocyte ratio*											
US: *n* = 634 (ADNI)	72 ± 7	43%	0%	7%	35%	3.0T	n.a.		–	–	(88)
Asia: *n* = 3052 (PRECISE study)	61 ± 7	43%	3%	6%	10%	3.0T	–	–	–	–	(89)
Asia *n* = 879	63 ± 12	62%	0%	n.a.	?	1.5T and 3.0T	↑	↑	n.a.	–	(90)
Asia: *n* = 2,875 (health checkup)	56 ± 9	22%	0%	9%	4%	1.5T	n.a.	↑	–	–	(91)
Asia: *n* = 950 (health checkup)	66 ± 9	57%	0%	16%	n.a.	1.5T	n.a.	–	–	n.a.	(92)
*Neutrophils*											
US: *n* = 634 (ADNI)	72 ± 7	43%	0%	7%	35%	3.0T	n.a.	↑	**–**	**–**	(88)
Asia: *n* = 3052 (PRECISE study)	61 ± 7	43%	3%	6%	10%	3.0T	↑	**–**	↑	**–**	(89)
*Monocytes*											
US: *n* = 634 (ADNI)	72 ± 7	43%	0%	7%	35%	3.0T	n.a.	↑	**–**	**–**	(88)
EU: *n* = 51 (RUN DMC)	70 ± 6	88%	0%	24%	49%	1.5T	n.a.	↑	n.a.	n.a.	(86)
*T cells*											
EU: n = 61	64 ± 8	100%	0%	10%	18%	1.5T	**↓**	n.a.	n.a.	n.a.	(93)
*TNFα*											
EU: *n* = 268 (MEMO study)	72	60%	8%	15%	n.a.	1.5T	n.a.	**–**	**–**	n.a.	(94)
Asia: *n* = 137	65 ± 8	62%	0%	n.a.	n.a.	1.5T	n.a.	**–**	n.a.	n.a.	(95)
Asia: *n* = 117	65 ± 8	34%	12%	n.a.	n.a.	3.0T	↑	n.a.	n.a.	n.a.	(96)
EU: *n* = 51 (RUN DMC)	70 ± 6	88%	0%	24%	49%	1.5T	n.a.	**–**	n.a.	n.a.	(86)
*IL-1β*											
EU: *n* = 268 (MEMO study)	72	60%	8%	15%	n.a.	1.5T	n.a.	**–**	**–**	n.a.	(94)
Asia: *n* = 117	65 ± 8	34%	12%	n.a.	n.a.	3.0T	**–**	n.a.	n.a.	n.a.	(96)
EU: *n* = 51 (RUN DMC)	70 ± 6	88%	0%	24%	49%	1.5T	n.a.	**–**	n.a.	n.a.	(84)
*IL-6*											
Asia: *n* = 117	65 ± 8	34%	12%	n.a.	n.a.	3.0T	↑	n.a.	n.a.	n.a.	(96)
EU: *n* = 1841 (3C study)	73 ± 4	43%	2%	9%	n.a.	1.5T	n.a.	↑	**–**	n.a.	(97)
EU: *n* = 51 (RUN DMC)	70 ± 6	88%	0%	24%	49%	1.5T	n.a.	↑	n.a.	n.a.	(86)
EU: *n* = 268 (MEMO study)	72	60%	8%	15%	n.a.	1.5T	n.a.	**–**	**–**	n.a.	(94)
EU: *n* = 634 (Lothian BC study)	73 ± 1	49%	7%	n.a.	n.a.	1.5T	n.a.	**–**	n.a.	n.a.	(98)
Asia: *n* = 137	65 ± 8	62%	0%	n.a.	n.a.	1.5T	n.a.	**–**	n.a.	n.a.	(95)
Asia: *n* = 194	67 ± 8	66%	0%	21%	n.a.	1.5T	n.a.	n.a.	↑	n.a.	(99)
Asia: *n* = 97	66 ± 8	?	0%	45%	n.a.	1.5T	n.a.	n.a.	↑	n.a.	(100)
Asia: *n* = 431	69 ± 9	71%	0%	36%	15%	1.5T	n.a.	n.a.	n.a.	↑	(101)
Asia: *n* = 201	64	47%	0%	41%	24%	3.0T	n.a.	n.a.	n.a.	↑	(102)
*IL-10*											
EU: *n* = 268 (MEMO study)	72	60%	8%	15%	n.a.	1.5T	n.a.	**–**	**–**	n.a.	(94)
EU: *n* = 51 (RUN DMC)	70 ± 6	88%	0%	24%	49%	1.5T	n.a.	**–**	n.a.	n.a.	(86)
*IL-17*											
EU: *n* = 51 (RUN DMC)	70 ± 6	88%	0%	24%	49%	1.5T	n.a.	**–**	n.a.	n.a.	(86)
Asia: *n* = 117	65 ± 8	34%	12%	n.a.	n.a.	3.0T	↑	n.a.	n.a.	n.a.	(96)
*IL-18*											
Asia: *n* = 431	69 ± 9	71%	0%	36%	15%	1.5T	n.a.	n.a.	n.a.	↑	(101)
*PDGFRβ*											
Asia: *n* = 158(CSF)	63 ± 7	n.a.	0%	n.a.	n.a.	3.0T	**–**	n.a.	n.a.	n.a.	(103)
*sTREM2*											
Asia: *n* = 66(plasma)	66 ± 11	79%	100%	53%	>68%	3.0T	n.a.	↑	**–**	**–**	(84)
*[^11^C]PK11195 PET imaging*											
EU: *n* = 42	72 ± 8	24%	0%	45%	36%	3.0T	↑	↑	**–**	↑	(104)

Summary of human studies that assessed associations of systemic immune markers and neuroimaging markers of cerebral small vessel disease (CSVD), including white matter hyperintensities (WMH), lacunar infarcts (LI), and cerebral microbleeds (CMB), quantified from magnetic resonance imaging (MRI). Immune markers include neutrophil-to-lymphocyte ratio, frequencies of neutrophils, monocytes, T cells, and levels of cytokines [in blood plasma via enzyme-linked immunosorbent assays or for the Radboud University Nijmegen Diffusion tensor and Magnetic resonance imaging Cohort (RUN DMC) study ex vivo production capacity of circulating peripheral blood mononuclear cells], such as tumor necrosis factor α (TNFα), interleukin 1β (IL-1β), IL-6, IL-10, IL-10, IL-17, IL-18. Microglial activation markers include soluble triggering receptor expressed on myeloid cell 2 (sTREM2) levels and translocator protein [11C]PK11195 positron emission tomography (PET) imaging. Associations are visualized for CSVD severity sum scores (composites of WMH, LI, and CMB) and separately for WMH, LI, and CMB according to the provided data from original studies (positive: **↑**, negative: **↓**, nonsignificant: **–**). In addition, we present prevalence for arterial hypertension (HTN), and for LI and CMB, which indicate CSVD severity. ADNI, Alzheimer’s Disease Neuroimaging Initiative; CSF, cerebrospinal fluid; EU, Europe; n.a., not available; Lothian BC, Lothian birth cohort 1936; MEMO, The Memory and Morbidity in Augsburg Elderly; PDGFRβ, platelet-derived growth factor receptor β; T, Tesla; US, United States of America.

**Table 2.** Relationship between systemic immune markers and longitudinal changes of neuroimaging markers of cerebral small vessel disease

Study	ΔFU	Mean Age	% HTN	% Stroke	% LI	% CMB	MRI	∼CSVD	∼WMH	∼LI	∼CMB	Reference
*Neutrophil-to-lymphocyte ratio*												
US: *n* = 634 (ADNI)	4 yr	72 ± 7	43%	0%	7%	35%	3.0T	n.a.	↑	-	-	(88)
*Neutrophils*												
US: *n* = 634 (ADNI)	4 yr	72 ± 7	43%	0%	7%	35%	3.0T	n.a.	↑	**-**	**-**	(88)
*Monocytes*												
US: *n* = 634 (ADNI)	4 yr	72 ± 7	43%	0%	7%	35%	3.0T	n.a.	↑	**-**	**-**	(88)
EU: *n* = 51 (RUN DMC)	9 yr	70 ± 6	88%	0%	24%	49%	1.5T	n.a.	↑	n.a.	n.a.	(86)
*TNFα*												
EU: *n* = 51 (RUN DMC)	9 yr	70 ± 6	88%	0%	24%	49%	1.5T	n.a.	**-**	n.a.	n.a.	(86)
*IL-1β*												
EU: *n* = 51 (RUN DMC)	9 yr	70 ± 6	88%	0%	24%	49%	1.5T	n.a.	**-**	n.a.	n.a.	(86)
*IL-6*												
EU: *n* = 51 (RUN DMC)	9 yr	70 ± 6	88%	0%	24%	49%	1.5T	n.a.	↑	n.a.	n.a.	(86)
*IL-10*												
EU: *n* = 51 (RUN DMC)	9 yr	70 ± 6	88%	0%	24%	49%	1.5T	n.a.	**-**	n.a.	n.a.	(86)
*IL-17*												
EU: *n* = 51 (RUN DMC)	9 yr	70 ± 6	88%	0%	24%	49%	1.5T	n.a.	↑	n.a.	n.a.	(86)
*sTREM2*												
US: *n* = 426 (ADNI, CSF)	2 yr	72 ± 7	45%	0%	31%	26%	3.0T	↑	↑	**-**	↑	(85)

Summary of human studies that assessed associations of systemic immune markers and longitudinal progression of cerebral small vessel disease (CSVD), including changes in white matter hyperintensity (WMH) volume, incident lacunar infarcts (LI), and incident cerebral microbleeds (CMB), quantified from magnetic resonance imaging (MRI). Immune markers include neutrophil-to-lymphocyte ratio frequencies of neutrophils, monocytes, and levels of cytokines (via enzyme-linked immunosorbent assays in blood plasma or after ex vivo production capacity of circulating peripheral blood mononuclear cells), such as tumor necrosis factor α (TNFα), interleukin 1β (IL-1β), IL-6, IL-10, IL-10, IL-17. Soluble triggering receptors expressed on myeloid cell 2 (sTREM2) levels refer to microglial activation. Associations are visualized for CSVD severity sum scores (composites of WMH, LI, and CMB), and separately for WMH, LI, and CMB according to the provided data from original studies (positive: **↑**, negative: **↓**, nonsignificant: **-**). In addition, we present baseline prevalences for arterial hypertension (HTN), and for LI and CMB, which indicate CSVD severity. ADNI, Alzheimer’s Disease Neuroimaging Initiative; CSF, cerebrospinal fluid; EU, Europe; ΔFU, median follow up; n.a., not available; Lothian BC, Lothian birth cohort 1936; MEMO, The Memory and Morbidity in Augsburg Elderly; RUN DMC, Radboud University Nijmegen Diffusion tensor and Magnetic resonance imaging Cohort; T, Tesla; US, United States of America.

There is large heterogeneity and inconsistencies in the human cohort data itself or in comparison to the hypothetical framework we have presented. Most of the studies have included subjects in early late-life with, presumably, longer-lasting hypertension, which also becomes evident through some amount of advanced CSVD pathology (i.e., cerebral microbleeds, lacunar infarcts (LI), more pronounced WMH). At the same time, a substantial proportion of study participants seem to have not suffered from arterial hypertension despite revealing markers of CSVD pathology. This means, that, for example, WMH, might have been caused by probably concurrent nonhypertensive CSVD or nonCSVD conditions. And, likewise, usually only part of the study population displayed CSVD pathology, pointing out that diagnostic heterogeneity could underlie data heterogeneity. In case CSVD severity and extent have been given—which has not consistently been the case—only markers indicative of more advanced disease were specified. Neither more early hypertensive brain pathologies, such as CBF alterations, nor the full spectrum of CSVD markers, such as microinfarcts or PVS, have been denoted (see Ref. 110). The relationship between immune markers, hypertension or CSVD, and cognitive function has been taken into account by none of the existing studies yet.

Based on the existing data, new questions—and challenges—arise for future translational studies in humans:

1)  What are the best-suited human cohorts that we should focus on when aiming at the translational understanding of immune signatures in early hypertensive disease stages?

There is a need for studies toward initial hypertensive disease stages, which—presumably—could be best captured already in midlife. In that instance, the focus should be on population-based data from large cognitively unimpaired cohorts without—or at most just subtle—CSVD pathology. The Radboud University Nijmegen Diffusion Tensor and Magnetic Resonance Cohort (RUN DMC) study, for example, has been implemented to address questions on the relationship between CSVD and cognitive impairment. However, most participants have already entered early-late life, have some degree of more pronounced CSVD and, there is a significant proportion of subjects without arterial hypertension [Tables 1 and 2; and see for example, Ref. (111)]. In addition, longitudinal approaches are warranted to uncover temporal relationships or certain dynamics of immune system activation. For example, in neurodegenerative diseases, there is commonly a biphasic immunological response of the central nervous system, and one study in experimental hypertension just recently pointed toward similarly dynamic microglial activation profiles, as outlined above (38, 112, 113).

2)  Which markers from which methods should we focus on?

We have pointed to the pivotal importance of the innate and adaptive immune system or the NVU and its secretome, with complex relationships and interactions between the different players. From a translational point of view, deep immunophenotyping in biofluids emerges as a promising tool, measuring pericyte (sPDGFRβ) or further NVU cell-type specific injury [for example, through extracellular vesicles (114)], frequencies of immune cell subpopulations and proinflammatory (e.g., IL-1β, IL-6, IL-17, INF-γ) or anti-inflammatory (e.g., IL-10) cytokines. Awareness has to be taken of the source of immune markers, either from the periphery or the central nervous system. Particularly in the case of BBB integrity loss, blood markers might not be able to distinguish between these different sources. Overall, the combination of immunophenotyping with advanced neuroimaging of the BBB and brain clearance routes will even increase the potential to disentangle inflammatory endotypes at the neurovascular-immune interfaces in hypertension (115).

2)  How do we have to deal with brain-disease-related phenotypic heterogeneity in arterial hypertension?

There has been upcoming evidence showing that different clinical phenotypes and downstream pathologies in (hypertension-related) CSVD mirror different advancements of the disease progress, which impacts the degree and stage of immune system activation. Hence, the cognitively impaired phenotype or existence of (mainly) ischemic brain disease (e.g., less pronounced WMH) seems to indicate less advanced CSVD compared with a clinical presentation with symptomatic (hemorrhagic) stroke or existence of hemorrhagic brain disease (e.g., cerebral microbleeds) (116, 117). CSVD subtypes have thus consequently been considered separately, especially when it comes to the question of endotypes and temporal evolution of the activated immune system.

3)  And which role does the existence of hypertension-related diseases of other organs play?

There is no isolated effect of arterial hypertension on the brain. Instead, several parts of the body are affected as well, and, for example, the heart should be mentioned here. The SNS and immune system take part in the—presumably—blood-pressure-independent communication between these involved body parts, for example, the communication along the brain-heart or heart-brain axis (118). Furthermore, there are, to give another example, multidirectional interactions between psychological factors, such as chronic psychosocial stress, brain function, hypertension, and the activated immune system (119). These aspects will provide great challenges and will have to be taken into account when we aim to understand the unique relationship between hypertension and immune system activation.

To conclude, we here highlight the role of presumably sequential inflammatory responses in hypertension pathogenesis for microvascular brain pathology. However, proof and translational validation of long-term immunological adaptation mechanisms and dynamics in human arterial hypertension and its impact on cognitive impairment are still missing. Particularly during the last decade, a huge understanding has emerged about the pivotal role of immune system activation in neurodegenerative disorder initiation and progression. Arterial hypertension and CSVD research will rapidly follow the same line. In that sense, hypotheses derived from experimental research, together with innovative biofluid and neuroimaging technologies, will let us move forward in understanding and targeting the immune system in hypertension as one of the leading threats to cognitive health.

## GRANTS

This work was supported by the Open Access Publication fund of medical faculty of the Otto-von-Guericke-University Magdeburg.

## DISCLOSURES

No conflicts of interest, financial or otherwise, are declared by the authors.

## AUTHOR CONTRIBUTIONS

P.A. and S.H. prepared figures; S.S., P.A., and S.H. drafted manuscript; S.S., P.A., L.M., A.P.G., P.M., KN., H.M., M.D., J.B., S.V., S.G.M., I.R.D., A.D., and S.H. edited and revised manuscript; S.S., P.A., L.M., A.P.G., P.M., K.N., H.M., M.D., J.B., S.V., S.G.M., I.R.D., A.D., and S.H. approved final version of manuscript.
